# Recent Progress in Treating Protein–Ligand Interactions with Quantum-Mechanical Methods

**DOI:** 10.3390/ijms17050742

**Published:** 2016-05-16

**Authors:** Nusret Duygu Yilmazer, Martin Korth

**Affiliations:** Institute for Theoretical Chemistry, Ulm University, Albert-Einstein-Allee 11, 89069 Ulm, Germany; duygu.yilmazer@uni-ulm.de

**Keywords:** protein/ligand interactions, quantum chemistry, density functional theory, semi-empirical quantum mechanical methods

## Abstract

We review the first successes and failures of a “new wave” of quantum chemistry-based approaches to the treatment of protein/ligand interactions. These approaches share the use of “enhanced”, dispersion (D), and/or hydrogen-bond (H) corrected density functional theory (DFT) or semi-empirical quantum mechanical (SQM) methods, in combination with ensemble weighting techniques of some form to capture entropic effects. Benchmark and model system calculations in comparison to high-level theoretical as well as experimental references have shown that both DFT-D (dispersion-corrected density functional theory) and SQM-DH (dispersion and hydrogen bond-corrected semi-empirical quantum mechanical) perform much more accurately than older DFT and SQM approaches and also standard docking methods. In addition, DFT-D might soon become and SQM-DH already is fast enough to compute a large number of binding modes of comparably large protein/ligand complexes, thus allowing for a more accurate assessment of entropic effects.

## 1. Introduction and Overview

The accurate and robust computational prediction of the relative strength of protein/ligand interactions is at the heart of virtual drug design [1,2]. A computational strategy that is able to evaluate protein/ligand binding affinities reliably and quickly over a wide range of protein targets and drug compounds is, unfortunately, still elusive [3,4,5]. This problem stems from the fact that free energies of binding for large molecular systems have to be computed to very high accuracy. Enthalpic contributions can now be covered, even at the quantum mechanical level, but the estimation of entropic effects is still complicated at all levels of theory [6,7]. A range of algorithms are available to either estimate or compute these contributions from classical molecular dynamics (MD) simulations [8], but a number of recent studies have shown that the accuracy of the underlying potential energy hypersurfaces is often at least questionable [9,10]. This is problematic, because very high accuracies are needed, and delicate enthalpy/entropy compensation effects have to be taken into account [11]. If quantum mechanical methods are applied, the problem lies in identifying the most important binding modes, as the number of modes that can be subjected to calculations is limited by computing power. In the past, researchers had to choose between either covering the dynamics of the system at hand while relying on very approximate classical (molecular mechanics, MM) energy models, or using a highly accurate energy model for a single binding mode, thus effectively ignoring the dynamics of the problem. It should be noted that thorough investigations of the role of entropic contributions are of course only possible with accurate energy models, and thus critically rely on the availability to do quantum mechanical calculations on a large number of binding modes, at least for model systems [12,13].

More advanced approaches can, on the other hand, build upon existing models. Sophisticated algorithms are available for the identification of possible binding modes—*i.e.*, the “posing” step of standard “docking” procedures. These seem to work rather well, unlike the evaluation of binding energies—*i.e.*, the “scoring” step [14]. Alternatively, snapshot structures can be extracted from classical molecular dynamics simulations [8,15]. A very interesting solution was put forward by Zheng *et al.* with their “movable type” method [16]. First, interaction pairs are constructed, then two tables with interaction strengths and interaction probabilities are constructed, and binding free energies are finally computed by applying statistical mechanics using these two tables. The same approach was developed in the past by Klamt for describing liquid phase properties with his COSMO-RS theory [17]. The most attractive features of this approach are that no explicit sampling is necessary and that pair-wise interactions can be computed at arbitrary levels of theory.

Free energies of binding can thus be calculated with quantum mechanical approaches from weighted averaging over free energies of pre-selected binding modes (or pair-wise interactions), taking into account the entropic effects of having a range of modes accessible for the system [18]. For details on ensemble averaging calculations in the context of non-covalent interactions and free energies of solvation, see recent reviews by Gilson [6] and Jensen [19]. The most important point is that entropic contributions are split into a part that can be computed for single binding modes using normal mode analysis and a part that stems from the fact that the system can change between single binding modes and which is related to the relative energies of these binding modes. While dispersion-corrected density functional theory (DFT-D) methods are still too slow for normal mode analysis of large biomolecules, dispersion and hydrogen bond-corrected semi-empirical quantum mechanical (SQM-DH) methods now allow for such calculations [20]. Free energies of the single binding modes can be computed within standard rigid-rotor, harmonic-oscillator approximations from energy and vibrational frequency calculations of the complex, protein, and ligand structures. An excellent overview of computing binding free energies of host–guest and similar systems in aqueous solution was given by Jensen [19], who additionally discusses possible error sources, including the role of explicit solvation and counter-ions. One advantage of quantum mechanical (QM) level approaches over classical methods is the correct treatment of polarization effects. This has the important side-effect that (continuum) solvation model results are usually already much improved for SQM, but especially for DFT methods [19]. “Dispersion corrected” density functional theory (DFT-D) methods have now become the *de facto* standard for larger molecular systems that are dominated by non-covalent interactions like protein/ligand complexes [21,22]. DFT-D was shown to perform as accurately as high-level quantum chemistry methods for such problems and is routinely applicable to systems with several hundreds of atoms. Uncorrected DFT does not account for London dispersion (dispersive van-der-Waals) interactions, which became a problem of the utmost importance when larger bio and also nano-structures came into reach during the late 1990s. The systematic application of dispersion corrections terms by Stefan Grimme [21,22] turned out to be a very efficient way of dealing with this problem. DFT methods are not yet fast enough for overall free energy calculations of more than model systems, but they can serve as a reference for faster methods, and they can give important insight into the balance of interactions, especially also concerning the interplay of enthalpic and entropic parts.

Very recently, dispersion- and also hydrogen-bond (H)-corrected semi-empirical quantum mechanical (SQM-DH) methods were developed, which perform almost as accurately as DFT-D for non-covalent interactions [23,24]. SQM-DH methods are about three orders of magnitude faster than DFT-D, which makes them very promising candidates to compute accurate free energies of binding for protein/ligand systems. In the following we will give an overview of recent developments in the field of applying SQM-DH, DFT-D, and higher-level quantum chemistry methods to protein/ligand systems or related model systems. A larger number of older (semi-empirical) quantum mechanical studies are available, but they usually apply “uncorrected” DFT or SQM methods [14,25], which are now known to perform very unreliably for non-covalent interactions, and are therefore not reviewed here. Finally, it needs to be mentioned that the overall problem of designing new drugs is of course a much more complex one. Ideas for the working mechanism of specific drugs have to be developed, target proteins have to be identified, structural information about proteins has to be collected, and input has to be prepared so that computational models can be applied. The problem of how to arrive at good model systems for calculations can easily become as complicated as actually performing calculations. However, similar to the case of enthalpic and entropic contributions, thorough investigations of error sources again have to be based on the application of trustworthy energy models.

## 2. Dispersion-Corrected Density Functional Theory (DFT-D) and Higher-Level Quantum Chemistry Investigations

### 2.1. Host/Guest and Other Model Systems

Most DFT-D and higher-level wave function theory (WFT)-based work in the field has been done on model systems and especially host/guest complexes, which are like protein/ligand complexes dominated by non-covalent interactions. The Grimme group has published not only a large number of studies on the role of dispersion interactions in chemistry (see for instance [26]), but has also produced a number of very thorough studies on how to compute host/guest interaction energies in a reliable fashion. Concerning electronic energies, Antony *et al.* [27] benchmarked generalized gradient approximations (GGA) and hybrid DFT-D3 methods against approximate Coupled Cluster data at the LPNO-CEPA-1/CBS (local pair natural orbital coupled electron pair approximation/complete basis set) level for protein/ligand model systems and found typical errors of 1–2 kcal (or 5%) with respect to theoretical reference data and 2–4 kcal/mol (or 10%) with respect to the experimental references. Notably, second order Møller–Plesset perturbation method (MP2) was found to perform less accurately and slower than DFT-D methods. Antony and Grimme [28] also showed how DFT-D3 can be applied to complexes of up to a few thousand atoms via molecular fragmentation with conjugated caps (MFCC). For MFCC (further discussed in references [29,30,31]), the protein is decomposed into fragments, which are then capped with typical end groups. MFCC is found to work well for the artificial case of a neutral peptide, but convergence problems are found for fragments with multiple charges. Grimme [12] then turned to go from enthalpies to free energies of binding for host/guest systems, and found similar overall errors when additionally applying implicit solvation models and lower-level estimates for the dynamical terms. Further studies from the Grimme group followed, an overview is given in Antony *et al.* [32]. They found that free energies of binding for host/guest systems can typically be computed up to an accuracy of about 2 kcal/mol if a single binding mode is dominating, and emphasize the critical role of dispersion interactions, so that either DFT-D with triple-zeta basis sets or Coupled Cluster (rather than MP2) with very large basis set need to be employed.

Skylaris and co-workers follow a somewhat complementary route: they perform “linear-scaling” plain-wave (periodic boundary condition) DFT calculations, which in principle allows them to go to very large system sizes, but limits the applicability of many existing techniques from computational chemistry [33,34,35,36]. However, they also work on using electrostatic embedding techniques, for instance [33]. Cole *et al.* [34] investigate protein/protein interactions, which are of fundamental importance to biological processes. Protein/protein interactions are also a very good example of when periodic DFT calculations might become advantageous, as the relevant systems cannot be easily broken down into smaller “cluster-based” calculations. Looking at the interaction of the breast cancer suppressor BRCA2 with RAD51, they want to understand how the former binds and delivers the latter to sites of DNA damage for repair. The lack of experimental structural information is addressed with model system calculations at the force field and DFT-D level. Periodic DFT calculations are of course no remedy for the problem of how to take into account entropic contributions, and poor agreement with experiment is accordingly found in cases where these contributions matter [35]. Fox *et al.* [36] ran into the same problem when investigating a protein/model system of more than 2500 atoms for the interaction of eight small aromatic ligands with the T4-lysozyme double mutant L99A/M102Q protein polar binding site.

The Hobza group has published a large body of work on the accurate calculation of non-covalent interactions and the benchmarking of SQM *vs.* DFT *vs.* WFT methods for this purpose (see for instance reference [20]). For a number of model systems, theoretical references are now available up to a full configuration interaction level [37]. Other benchmark work includes the extensive sampling of the conformational space of nine HIV-1 protease inhibitors [38], where 1600 conformations of each ligand are investigated at the SQM-DH level, and errors from using single conformations and short molecular dynamics runs are about 3 kcal/mol. Further studies address the effects of using implicit solvent models and conformational ensembles [39], where the multiconfigurational COSMO-RS is found to perform best. For modeling protein/ligand interactions, Hobza and co-workers most often turn to SQM-DH methods, which is why this work is discussed in the following section.

Ryde and co-workers have published extensively on using QM/MM type approaches for treating protein/ligand interactions, but (S)QM methods in the older works “uncorrected” QM methods and these studies will accordingly not be referenced here. One older investigation was using MP2 [40], which again cannot be recommended for non-covalent interactions. Newer work is partly based on “enhanced” methods and also very high-level WFT approaches. Mikulskis *et al.* [41] for instance computed free energies of binding for nine cyclic carboxylate guest molecules to an octa-acid host. The best agreement with experiment was found at the classical level. Results were worse with DFT, and again worse with their linearized coupled-cluster theory with single and double excitations LCCSD (T0) approximate Coupled Cluster approach. Andrejic *et al.* arrived at LCCSD (T0)/CBS calculations for a 200 atom host/guest model system [42] by calculating short-range interactions pairwise at the QM level and treating long-range interactions with a polarizable multipole approach. This approach gives an accuracy of 2–3 kcal/mol; *i.e.*, comparable to a proper DFT-D treatment, but almost no correlation with experiment is observed, as only a single binding mode could be treated.

Protein/ligand-screenings beyond proof-of-concept studies on model systems [43,44,45,46,47,48,49] are not yet done with DFT or higher-level WFT methods, but most often SQM-DH methods—see the next section for details. It was nevertheless already shown that QM methods are advantageous for protein structure refinement [50,51,52,53]. Christensen *et al.* [54] were even able to use backbone amide proton chemical shifts calculated at the QM-level for this purpose [29,55].

### 2.2. Local Wave Function Theory Methods

A very interesting and important development for the future of high-level quantum mechanical calculations on large molecular systems (and therefore also protein/ligand complexes) is the advent of robust local coupled cluster (CC) methods, and especially local pair natural orbital (LPNO) approaches [56]. LPNO-CC methods lose very little (usally substantially less than 1.0 kcal/mol) upon canonical CC in terms of accuracy, but can be applied to systems that are normally only in the reach of DFT. Results furthermore converge systematically to the canonical CC ones if tighter thresholds are chosen, which of course also increases computational costs. LPNO-CC methods furthermore show an improved basis set convergence, leading to higher gains with larger basis sets, thus bringing several hundred atoms in reach of highly accurate quantum chemistry methods. The most imporant feature is that LNPO-CC methods can (unlike other local CC approaches) be applied to a wide range of problems in a black box fashion, which makes it in principle suitable for re-screening purposes. LPNO-CCSD [57] is based on earlier work on LNPO coupled electron pair approximation (CEPA) methods [58], and was then re-designed using pre-screening, truncation, and domain techniques, which lead to the DLPNO-CCSD method. The advantage of this ansatz to exploit locality for CC is that PNOs allow for very compact wave functions, which in turn allow for large and therefore reliable domain approximations. First benchmark calculations showed that DLPNO-CCSD performs very similarly to LNPO-CCSD and thus canonical CCSD, but becomes substantially faster for medium to large systems, as now CPU time, as well as RAM and disk space resources scale nearly linearly with system size.

Liakos *et al.* [59] thoroughly benchmarked DLPNO-CCSD(T) for a broad range of chemical applications. They found that default thresholds (three parameters overall) allow for an accuracy of 1 kcal/mol or better, while tighter thresholds can reduce errors to about 0.25 kcal/mol. Most importantly for the case of protein/ligand interactions, default thresholds already lead to average errors as small as 0.3 kcal/mol for non-covalent interactions. Excellent results are also achieved for linear *versus* folded conformations of unbranched alkanes [60], which could be considered as the simplest conceivable atomic-scale model system for protein folding. As in many cases, the CC step in DLNPO-CCSD is not more costly than the Hartree-Fock (HF) step; Liakos and Neese [61] benchmarked DLPNO-CCSD(T) against DFT methods with respect to both accuracy and computational cost. Even with loose thresholds, DLPNO-CCSD(T) is more accurate than any DFT approach, while computational costs for aug-cc-pVTZ basis sets are roughly similar to B3LYP. Errors below 1 kcal/mol are achieved with default thresholds, computation times become two times higher. CCSD(T)/CBS level accuracy is achieved with tight thresholds, while computation times are around only four times higher than B3LYP. The scaling of the new approach is furthermore like HF or DFT dominated by the self-consistent field equations, which is quadratic to cubic, but can be made linear scaling for large systems. Convergence with basis set size is nevertheless somewhat slower for DLNP-CCSD(T) than DFT. As several of the above-listed benchmark studies included model systems for biomolecular interactions, it seems very likely that the availability of fast and extremely accurate WFT methods will have a strong impact on the quantum-level re-scoring of protein/ligand interactions in the near future.

## 3. Dispersion and Hydrogen Bond-Corrected Semi-Empirical Quantum Mechanical (SQM-DH) Applications

### 3.1. Model Systems and Related Studies

A large number of studies have now shown that SQM-DH methods perform almost as accurately as DFT-D for a wide variety of systems that are dominated by non-covalent interactions [62,63,64,65,66,67,68,69,70,71,72,73,74,75]. Furthermore, detailed benchmark studies, in comparison to classical approaches, DFT-D, and highly accurate WFT data is available in the original development papers as well as in separate benchmark studies [76]. This includes investigations into hydrogen-bond cooperativity effects [77] and halogen-bonded systems [78]. Several SQM-DH models exist, but it is important to note that the main difference for non-covalent interactions comes from including DH correction, not the actual model one is using [79]. Methods include AM1-FS1 [80,81], PM6-DH2 [82], and PM6-DH+ [83]. PM6-DH2 and PM6-DH+ were further improved for stable molecular dynamics simulations, leading to PM6-D3H4 [84] and arguably the most advanced model, PM6-D3H+ [85]. See references [23,24] for details on SQM-DH methods. For complexes of hundreds of atoms, SQM-DH methods are fast enough for the calculation of hundreds of millions of configurations. PM6-DH+ for instance was applied for the calculation of 320 million interaction energies for a peptide/ligand system [86]. Excellent correlation with experiment was observed, with an *R*^2^ value of 0.9, in comparison to 0.7 for an classical approach. Like DFT-D, SQM-DH methods were shown to be valuable for structure refinement purposes [75,87,88,89,90]. Again, like DFT-D, SQM-DH proved to be an intesting tool for applications in materials science [71,91,92,93,94,95], giving some hope for tackling problems at the interface of life and materials science.

### 3.2. Host/Guest Systems and Protein/Ligand Interactions

#### 3.2.1. Host/Guest Systems

The most important model systems with relation to protein/ligand interactions are host/guest systems, for which SQM-DH methods are now routinely applied [12,96,97,98,99]. Muddana *et al.* calculated free energies of binding with their minima mining approach; *i.e.*, the weighted averaging over dominant binding modes from classical dynamics, for CB7 host/guest systems in very good agreement with experiment [13]. This study clearly shows the power of SQM-DH-based approaches, although massive upscaling is necessary to address real-life virtual drug design problems.

#### 3.2.2. Protein/Ligand Interactions

Such an upscaling as mentioned above is indeed possible, and is shown in studies from the Korth group for protein/ligand interactions [10,100]. The complete PDBbind2007 benchmark set is subjected to a systematic benchmark of MM, DFT, and SQM methods, and a set of smaller model systems is additionally checked with high-level WFT calculations. It is found that the accuracy first of all depends on the level of theory: SQM-DH methods perform very similarly to DFT-D and substantially better than MM methods. The performance for different methods at the same level of theory turned out to be rather similar. QM calculations can be restricted to smaller model systems of the full complexes without losing predictive capability, which brings all benchmark entries well into the reach of SQM-DH methods. Automatic model system preparation and the treatment of entropic effects are listed as major remaining challenges, though a more detailed analysis also revealed options for the further improvement of SQM-DH methods. An exhaustive comparison of WFT, DFT, and SQM methods with high-level reference data for the smaller complexes led the authors to recommend TPSS-D3(abc)/def-TZVPP as DFT method and PM6-DH+ as SQM-DH approach.

A series of docking studies from the Hobza group rely on SQM-DH methods with implicit solvation models and force field based entropic terms [63,101,102,103,104,105]. In these studies, SQM-DH agrees very well with DFT-D results and is found to be consistently better than classical methods, especially also for the identification of decoy structures.

A more systematic approach for the calculation of free energies of binding for protein/ligand systems was put forward by Stigliani *et al.* [106]. They apply SQM-DH methods on selected binding modes based on Autodock Vina results, and indeed find a good performance of Vina for the posing step, but the correct score only after SQM-DH re-optimization. Another systematic approach was published by Ucisik *et al.* [107]. Here, a special type of weighted averaging is applied, which the authors call “blurring”, and which arrives at ensembles of binding modes via a local sampling algorithm. Correlation between calculated and experimental data increases substantially with the use of SQM-DH methods. Further studies were published with SQM-DH level QM or QM/MM treatment of protein/ligand interactions [25,108,109,110,111,112,113].

## 4. Summary and Outlook

Recent years have seen increased activity in the field of improved virtual drug design models based on the QM-rescoring of classically-obtained binding mode ensembles. “Enhanced”, dispersion (D) and hydrogen-bond (H) corrected QM methods like DFT-D and SQM-DH are at the core of this “new wave” of QM-based scoring schemes. Unlike older DFT and SQM approaches, these methods perform very accurately for the non-covalent interactions that dominate protein/ligand binding. DFT-D shows an accuracy close to the most sophisticated (and much more costly) quantum chemistry approaches. SQM-DH methods are almost as accurate as DFT-D, but are additionally extremely fast, though potential hypersurface errors can lead to problems when optimizing highly-symmetric systems. DFT-D is now the *de facto* standard approach for large, non-covalent interaction-dominated systems, but SQM-DH and probably also other minimal (slower, but more robust) QM models like HF-3c [114] are an excellent choice for systems outside of the reach of DFT calculations. Such systems are, for instance, protein/protein interfaces as well as functional biomacromolecules and hybrid bio/nano-systems, at least when it comes to *in silico* design and optimization. Less attention needs to be paid to the actual DFT-D or SQM-DH method that is used. Fortunately, DFT-D methods are now readily available in standard quantum chemistry codes and PM6-DH2 as well as PM6-DH+ are available in MOPPAC [115]. Free D and DH codes are available for the implementation of new research ideas [116]. It seems very likely that DFT-D and SQM-DH methods will have a great impact on the development of more accurate approaches for treating protein/ligand interactions.
